# Exploring chronotype, sleep patterns, and health in petrochemical shift workers

**DOI:** 10.1016/j.aprim.2026.103480

**Published:** 2026-04-17

**Authors:** Hamidreza Mokarami, Somayeh Bolghanabadi, Reza Kazemi, Andrew Smith

**Affiliations:** a Department of Ergonomics, School of Health, Shiraz University of Medical Sciences, Shiraz, Iran; b Student Research Committee, Shiraz University of Medical Sciences, Shiraz, Iran; c Department of Occupational Health and Safety Engineering, School of Health, Shiraz University of Medical Sciences, Shiraz, Iran; d Department of Ergonomics, Shiraz University of Medical Sciences, Shiraz, Iran; e School of Psychology, Centre for Occupational and Health Psychology, Cardiff University, Iran

**Keywords:** Shift work, Sleep quality, Health disorders, Chronotype, Petrochemical workers, Trabajo por turnos, Calidad del sueño, Trastornos de salud, Cronotipo, Trabajadores petroquímicos

## Abstract

**Objective:**

Chronotype is a personal characteristic affecting an individual's adaptability to shift work. The present study aimed to investigate the relationship between chronotype, shift work-related health disorders and sleep quality in shift workers in the petrochemical industry in southern Iran.

**Design:**

This was a cross-sectional study.

**Site:**

The study was conducted in a petrochemical complex located in southern Iran.

**Participants:**

250 male shift workers with at least five years of shift work experience, no major physical or mental health issues, and no use of alcohol, drugs, or sleep medications.

**Interventions:**

No interventions were applied, as this was an observational cross-sectional study.

**Main measurements:**

Data were collected using the Shift Work Impact on Health and Social Disorders (SOS) Questionnaire, the Pittsburgh Sleep Quality Index (PSQI), the Morningness–Eveningness Questionnaire (MEQ), and the Cognitive Failure Questionnaire.

**Results:**

The prevalence of musculoskeletal, gastrointestinal, and cardiovascular disorders was significantly higher in morning types than evening types (*p* < 0.05). Cognitive failures and sleep quality also differed significantly between the two groups (*p* < 0.05).

**Conclusions:**

Evening-type individuals showed higher adaptability to shift work and lower vulnerability to negative consequences. Circadian typology should be considered when assigning workers to shifts, especially for sensitive jobs.

## Abstract (es)

**Objetivo:**

El cronotipo es una característica personal que afecta la adaptabilidad de un individuo al trabajo por turnos. El presente estudio tuvo como objetivo investigar la relación entre el cronotipo, los trastornos de salud relacionados con el trabajo por turnos y la calidad del sueño en trabajadores por turnos de la industria petroquímica en el sur de Irán.

**Diseño:**

Estudio transversal.

**Emplazamiento:**

El estudio se llevó a cabo en un complejo petroquímico ubicado en el sur de Irán.

**Participantes:**

250 trabajadores varones por turnos con al menos cinco años de experiencia en turnos, sin problemas graves de salud física o mental y sin consumo de alcohol, drogas ni medicamentos para dormir.

**Intervenciones:**

No se aplicaron intervenciones, ya que se trató de un estudio observacional transversal.

**Mediciones principales:**

Los datos se recopilaron mediante el Cuestionario de Impacto del Trabajo por Turnos en la Salud y Trastornos Sociales (SOS), el Índice de Calidad de Sueño de Pittsburgh (PSQI), el Cuestionario de Matutinidad-Vespertinidad (MEQ) y el Cuestionario de Fallos Cognitivos.

**Resultados:**

La prevalencia de trastornos musculoesqueléticos, gastrointestinales y cardiovasculares fue significativamente mayor en los tipos matutinos que en los vespertinos (p< 0,05). Los fallos cognitivos y la calidad del sueño también difirieron significativamente entre ambos grupos (p< 0,05).

**Conclusiones:**

Los individuos de tipo vespertino mostraron una mayor capacidad de adaptación al trabajo por turnos y una menor vulnerabilidad a las consecuencias negativas. Se sugiere considerar la tipología circadiana al asignar trabajadores a turnos, especialmente para trabajos más sensibles.

## Introduction

Shift work, defined as employment outside conventional daytime hours, is a common work pattern in industrial settings and affects approximately 20–25% of the workforce worldwide.^1^ Extensive evidence has shown that shift work is associated with an increased risk of occupational injuries, accidents, sleep disturbances, and a wide range of adverse health outcomes.^2^, ^3^ Despite numerous preventive strategies and organizational interventions, these negative consequences remain prevalent among shift workers, suggesting that current approaches may not adequately account for important individual-level factors influencing tolerance to shift work.^4^, ^5^, ^6^

Individual differences in circadian preferences, or chronotypes, represent a major source of variability in tolerance to shift work.^7^ Chronotype reflects an individual's intrinsic timing of sleep and wakefulness, largely determined by endogenous biological factors, and influences physiological and behavioral functions throughout the day.^7^, ^8^ Evidence suggests that misalignment between an individual's chronotype and their work schedule can exacerbate sleep disturbances, fatigue, and other health risks, highlighting chronotype as a critical factor to consider in the design and management of shift work systems.^9^, ^10^

Human chronotypes are commonly classified into morning (morningness) and evening (eveningness) types, reflecting intrinsic differences in circadian timing.^7^, ^11^ Morning types typically wake and sleep earlier, exhibiting an advanced phase of circadian rhythms, while evening types prefer later sleep and activity schedules, showing a delayed circadian phase, including shifts in melatonin secretion.^12^ These differences influence individuals’ adaptability to imposed work schedules, as misalignment between an individual's chronotype and their work hours can lead to sleep disturbances, fatigue, and reduced performance.^8^, ^13^ Extreme morning or evening types may face particular challenges in adjusting to shift work, highlighting the relevance of chronotype in planning and managing work schedules.

Several studies have examined the relationship between chronotype and various outcomes in shift work. Evening types generally report poorer sleep quality and higher daytime sleepiness compared to morning types.^14^, ^15^ Though some studies found no significant differences in sleep duration or performance.^13^, ^16^ Similarly, while certain research suggests associations between chronotype and increased risk of metabolic disorders, gastrointestinal issues, or decreased alertness during specific shifts,^17^, ^18^ other studies did not observe consistent effects on accident rates or overall health outcomes.^19^ These inconsistencies may result from variations in occupational settings, shift schedules, and outcome measures, highlighting the need for context-specific investigations. Collectively, the evidence indicates that chronotype is an important individual factor influencing adaptation to shift work, but the magnitude and nature of its impact remain unclear, particularly in industrial settings with slow rotation shift schedules.

In the South Pars petrochemical industries, employees work slow-rotation shifts due to the remote location of the facilities. These shifts involve regularly working outside conventional daytime hours (8:00 a.m. to 6:00 p.m.), such as seven days of day shifts, seven days of night shifts, followed by seven days off. Previous research has reported a high prevalence of shift work-related health issues in these industries, including sleep disturbances, gastrointestinal problems, and cardiovascular disorders.^20^ While prior studies have examined the effects of chronotype on sleep and health outcomes, most were conducted in non-shift work populations or focused on specific shift schedules. Accordingly, the present study aims to investigate the associations between chronotype, sleep quality, and health-related outcomes among shift workers in South Pars petrochemicals, providing context-specific evidence to inform shift work management.

## Materials and methods

The present cross-sectional study was conducted in a petrochemical complex in southern Iran, employing a total of 1535 male workers. All employees’ medical records at the occupational health center were systematically screened based on predefined inclusion criteria: at least five years of experience in the shift work system, absence of major physical or mental health problems, no history of alcohol consumption or drug addiction, and no use of sleep medications. Given the small number of workers reporting alcohol consumption or use of sleep medications, and considering the known effects of these substances on sleep patterns and circadian regulation, these individuals were excluded to reduce uncontrolled confounding and improve the interpretability of chronotype-related associations. Based on this screening, 510 employees were identified as eligible. Among them, 250 consented to participate, and their data were processed for the final analysis. For some variables, missing values were present and were treated as missing in the statistical analyses without any imputation. Therefore, all available data for each variable were used in the corresponding analyses, and the total number of participants may vary across columns in the tables.

All participants received a comprehensive explanation of the study objectives and procedures, and confidentiality was ensured through collective data analysis and anonymous questionnaires. Written informed consent was obtained from all participants, and data were collected in person.

### Tools

A structured questionnaire was used to assess socio-demographic characteristics and work-related variables, including age, education level, marital status, work experience, and shift type, as potential confounders. The SOS was employed to assess the effects of shift work on health and social outcomes; this instrument has been validated and culturally adapted for Persian-speaking populations.^6^ Sleep quality was measured using the Pittsburgh Sleep Quality Index (PSQI), which has demonstrated reliability and validity in both international and Persian contexts.^8^ The Morningness–Eveningness Questionnaire (MEQ) is a 19-item self-report instrument developed by Horne and Östberg to assess individuals’ circadian type based on sleep–wake timing and activity preferences,^21^ In this study, the validated Persian version of the MEQ was used. Based on previous Persian-language validation studies, participants were categorized into three chronotypes using the following cut-off points: morning type (scores ≥ 59), intermediate type (scores 42–58), and evening type (scores ≤ 41). The validity and reliability of the Persian version have also been confirmed in prior studies,^22^, ^23^ and cognitive failure questionnaire were used to examine cognitive failures.^24^

### Statistical analysis

The descriptive analysis provided statistics about the participants’ socio-demographic characteristics. First, an independent *t*-test compared the demographic characteristics, sleep quality variables and cognitive failures across chronotype types. Secondly, Chi-square tests examined the relationships between chronotype and five work system-related dimensions: shift satisfaction, effect on family life, digestive effects, cardiovascular effects, and musculoskeletal effects. The significance level was set at *p* < 0.05.

## Results

The mean age of the participants (*n* = 250) was 38.21 ± 9.77 years (range: 22–50 years), and the work experience was 12.54 ± 9.77 years (range: 5–30 years); all participants were male. Based on the Morningness–Eveningness Questionnaire (MEQ), participants could be classified into three groups: morning-type, evening-type, and intermediate. In our sample, 78 employees were classified as morning-type and 152 as evening-type, and no participants fell into the intermediate group. Morning-type participants demonstrated a longer mean work experience compared to evening-type participants (Table 1).

**Table 1:** Comparison of participants’ demographic characteristics by circadian type.

Variable	Mean (SD)		*p*-Value
	Morning type (*n* = 78)	Evening type (*n* = 152)	
Age (y)	39.3 (10.4)	37.7 (9.4)	0.236
Work experience (y)	14.1 (8.0)	11.8 (8.3)	0.040
Work hours (h/day)	10.6 (1.8)	10.4 (2.0)	0.471

More than 60% of workers with eveningness and 58% of those with morningness were dissatisfied with their shift work systems. There was no significant difference between the two groups for this variable. In terms of the impact of the shift system on family life, the morning types reported more problems, with nearly 61% of them stating that the work system harmed their family life. In contrast, 41% of the evening types reported a negative impact on family life. In terms of health, morning types reported more problems, with 82%, 51%, and 26% of these employees stating that they suffered from gastrointestinal, musculoskeletal, and cardiovascular problems. Among the evening types, 62%, 22%, and 5.2% had gastrointestinal, musculoskeletal, and cardiovascular issues (Table 2).

**Table 2:** Comparison of health outcomes between morningness and eveningness circadian types.

Variable	*N* (%)		*p*-Value
	Evening type	Morning type	
*Shift satisfaction*			
No	47 (58.0)	102 (60.4)	0.725
Yes	34 (42.0)	67 (39.6)	

*Family effect*			
No	32 (39.5)	99 (58.6)	0.005
Yes	49 (60.5)	70 (41.4)	

*Digestive effect*			
No	31 (38.3)	25 (14.8)	<0.001
Yes	50 (61.7)	144 (85.2)	

*Cardiovascular effect*			
No	79 (97.5)	125 (74.0)	<0.001
Yes	2 (2.5)	44 (26.0)	

*Musculoskeletal effect*			
No	63 (77.8)	83 (49.1)	<0.001
Yes	18 (22.2)	86 (50.9)	

The morning types had worse sleep quality and more cognitive failures than evening types, and there was a significant difference between the two groups. Table 3 presents the results for these two variables.

**Table 3:** Comparison of sleep quality and cognitive failures between morningness and eveningness circadian types.

Variable	Mean (SD)		*p*-Value
	Evening type	Morning type	
PSQI^a^	3.9 (2.7)	5.1 (2.8)	0.002
Cognitive failure	69.2 (18.1)	74.9 (16.2)	0.018

a PSQI = Pittsburgh Sleep Quality Index.

## Discussion

The widespread individual differences between employees are a leading challenge in planning shift work systems. As it limits the development of a uniform and adaptable schedule for all individuals.^7^ The circadian type or chronotype is considered one of the most significant individual differences.^25^ Therefore, the present study aimed to examine the association between chronotype and health disorders and sleep quality in shift workers at South Pars petrochemicals. Morning-type employees were observed to have a higher prevalence of cardiovascular, gastrointestinal, and musculoskeletal disorders compared to evening-type employees. These findings are generally consistent with those reported by Yu et al.^17^

There was no significant difference in the mean age of the morning and evening types. Age may act as a confounding factor in this type of research, as individuals tend to shift toward morningness with increasing age. The observed differences between chronotypes may reflect variations in adaptation to shift work schedules, although causality cannot be established from the present cross-sectional data. Previous research suggested that evening-type individuals may have higher rates of health disorders compared to the general population.^18^

There was no difference in the satisfaction of shift work in the two groups, with about half of the research population in both groups being not satisfied shift work. This result is consistent with other studies. Prior research indicates that individuals often prefer fixed shifts aligned with their chronotype, and misalignment may be associated with lower satisfaction and increased discomfort and fatigue.^13^, ^26^ Job dissatisfaction was high in both morning-type and evening-type employees, although evening-type workers were expected to report higher satisfaction due to their greater adaptability to shift work. This finding may be influenced by cultural and organizational factors in the Iranian work environment, such as high work pressure, long shift hours, social and family constraints, and misalignment of management systems with individual needs, which can contribute to elevated dissatisfaction in both groups.

Family life was another important factor in the study, and negative effects of shift work on this were higher in the morning types than the evening types. The observed association between work-family conflict and reported negative outcomes may reflect one of several factors contributing to these differences, although causal relationships cannot be determined from the cross-sectional design.^17^

The sleep quality of evening types was better than that of morning types. The results were consistent with a study by Ognianova et al.,^27^ but inconsistent with the results of Rique.^13^ The observed differences in sleep quality and quantity may be related to variations in circadian rhythm flexibility Additionally, evening-type employees may find it easier to sleep during the day, potentially due to the timing of melatonin secretion.^7^

The findings of the present study are partially consistent with previous research, although some discrepancies exist, which may be attributed to differences in the study population characteristics, industrial context, or assessment methods. The observed associations suggest that evening-type individuals were associated with better sleep quality and overall health, whereas morning-type individuals appeared to have a higher prevalence of adverse effects related to shift work.

Limitations of the current study include its focus on male workers from a single petrochemical complex and its cross-sectional design, which restricts the generalizability of the results and the ability to infer causal relationships. In addition, the exclusion of participants who reported alcohol consumption or use of sleep-inducing medications may have introduced selection bias and further limited the generalizability of the findings, as these individuals may differ systematically in chronotype and health characteristics compared to those included in the analysis. Moreover, only bivariate analyses were conducted in this study, which do not allow for controlling potential confounding factors such as age, years of work experience, or other relevant variables. Therefore, observed differences between chronotypes may not solely reflect their relationship with sleep quality or quality of life. Future studies should employ multivariate or regression analyses to more accurately examine the effects of chronotype while controlling for confounders. Future studies should consider larger and more diverse populations, including individuals who consume alcohol or use sleep-inducing medications, as well as workers from different industries, age groups, and genders, to more comprehensively assess the effects of shift work on health and performance.

Nevertheless, the findings have important practical implications: identifying employees’ chronotypes can inform shift scheduling and workforce selection, potentially mitigating health risks and enhancing job satisfaction, although further research is needed before broader clinical application. While this study is primarily relevant to occupational health, it also provides insights that can be useful for primary care and family medicine. Future research should expand the sample to include diverse industries, age groups, and genders, and consider longitudinal designs to examine the long-term effects of shift work on health and performance.

## Conclusion

The present study demonstrated that individuals’ chronotype is associated with their adaptability to shift work and the prevalence of health disorders. Employees with an eveningness chronotype exhibited greater capacity to adjust to shift schedules and were less susceptible to the negative consequences of shift work, whereas morningness individuals were more vulnerable to such adverse effects. Morningness participants reported higher prevalence of cardiovascular, gastrointestinal, and musculoskeletal disorders, along with poorer sleep quality and greater cognitive failures.

Based on these findings, it is recommended that employees’ chronotypes be considered when assigning shift work, particularly for safety-critical roles, with preference given to evening-type individuals. Such an approach may help reduce health risks and improve job satisfaction.

Nevertheless, the study had limitations, including its focus on male workers from a single petrochemical complex and its cross-sectional design, which restrict the generalizability of the results and the ability to infer causal relationships. Future research should include larger and more diverse samples across different industries, age groups, and genders, and employ longitudinal designs to examine the long-term effects of shift work on health and performance.

## Author contributions

H.M: Writing – review and editing, Writing – original draft, Conceptualization, Data curation. S.B: Writing – review & editing. A. P.S: Writing – review and editing, Writing – original draft. R.K: Supervision, Writing – review & editing, Conceptualization.

## Ethical considerations/Informed consent

The study was approved by the Scientific Committee and Medical Ethics of Shiraz University of Medical Sciences (IR.SUMS.REC.1395.147), and informed consent was obtained from all participants.

## Use of AI

We used AI-based tools to check grammar and refine the academic quality of the manuscript, although the primary writing was carried out by the authors.

## Funding

No funding was received.

## Conflict of interest

The authors declare no conflict of interest.

## Data availability

The original contributions presented in the study are included in the article/supplementary material, further inquiries can be directed to the corresponding authors.
